# Generalized 3D quasi-phase-matching model of image contrast in second harmonic generation microscopy of fibrillar collagen architectures

**DOI:** 10.1088/2515-7647/ae37b3

**Published:** 2026-01-30

**Authors:** Emily M Shelton, Paul J Campagnola

**Affiliations:** Department of Biomedical Engineering, University of Wisconsin-Madison, Madison, WI 53706, United States of America

**Keywords:** collagen, quasi-phasematching, fibril, emission pattern

## Abstract

Second harmonic generation (SHG) microscopy is a powerful tool in assessing collagen structure, especially with respect to differentiating the respective architectures of normal and diseased tissues. An under-explored area is exploiting SHG to determine the sub-resolution aspects of the collagen fibril size, polarity, and packing (∼50–100 nm diameters). Due to the phase-matching and associated coherence of SHG, these structural aspects are encoded in the wavelength dependence of the spatial emission and relative conversion efficiency, denoted the creation attributes. As a means to extract this information, we present a generalized 3D computational/theoretical treatment based on quasi-phase-matching (QPM), which can predict the SHG emission pattern and relative conversion efficiency using collagen models based on 3D biomimetic fibril architectures. Specifically, we incorporate random rather than purely periodic structures and non-ideal phase-matching $\left( {\Delta k \ne 0} \right)$ conditions. By exploration of parameter space, and comparison with imaging data, we can place bounds on the fibril architecture without the use of structural biology tools. The resulting predicted fibril sizes of real tissues are in good agreement with known values from electron microscopy. Moreover, by examining the role of heterogeneity, we have identified the contribution of small and large fibrils and clustering therein to the creation attributes, and the regimes where these dominate the spatial emission pattern. These simulations also resulted in good agreement with prior work on the wavelength dependence of SHG conversion efficiency, where the fibril size and packing are sufficient to reproduce experimental data without invoking a two-state model. This level of agreement provides validation of the model and also points to the need for this approach to treat the SHG responses due to the intrinsic complexity of many tissues.

## 1 Introduction

Over the last 25 years second harmonic generation (SHG) microscopy has emerged as a powerful tool for both visualizing and analyzing the collagen architecture in a wide variety of tissues. Adapting SHG into a microscopy technique provides great richness as it has intrinsic optical sectioning due to it being a nonlinear phenomenon, and can image into tissues to depths of a few hundred microns with a lateral resolution of ∼0.5 *µ*m, which is on the size scale of collagen fiber diameters [1, 2]. This has importance to human health as the collagen structure is remodeled in wide variety of diseased states [3]. Because of these attributes SHG microscopy has been used to study changes in collagen fiber organization in diseased tissues including cancers [4–7], fibroses [8–11], and connective tissue disorders [12–16]. However, the exploitation of the underlying physics offers significant opportunities to more fully examine collagen structure in tissues.

The fundamental requirements for SHG depend on the harmonophore, here collagen, to possess a permanent dipole to have nonzero first order hyper-polarizability, *β*, and assembling the molecules to have an overall alignment forming a non-symmetric environment to have non-vanishing second order nonlinear susceptibility, χ^(2)^ [17]. The underlying molecular properties of collagen that lead to the second order response have been investigated and shown that the nonlinearity arises from coherent addition of peptide bonds [18]. The specific aspects of the collagen molecule, chiral and achiral components that give rise to the nonlinearity have also been examined through the use of polarization analyses [19–22]. Analogous analyses have been used to probe details of the molecular structure and organization of the collagen and how it varies in diseased states [12, 23–25]. We have further used the creation attributes of the relative conversion efficiency and spatial emission pattern (or distribution of the forward, *F*_SHG_, and backward *B*_SHG_, emitted components) to characterize the sub-resolution fibril architecture and probe such changes therein between normal and diseased tissues [26, 27]. This can complement ultrastructural studies by electron microscopy that have shown the fibril sizes and distributions can be different in several diseases [28–30] as well as aging [31]. However, there is no complete theoretical treatment of SHG in the limit of non-ideal phase-matching in collagen architecture. This is a shortcoming as collagen fibril size, packing and polarity can be altered in diseased states, where these structural aspects are encoded in the spatial emission and relative conversion efficiency. Thus, a technique to probe these alterations on the ∼50–100 nm sizescale could provide valuable insight into disease etiology and progression. Here we focus on Col I as it is the primary isoform in many connective tissues including skin, tendon, bone, cornea and as well as stroma in many organs. Because of this ubiquity, it is important to study its fibrillar assembly and role in SHG contrast and is therefore the singular focus here.

We previously developed a heuristic model of SHG creation based on relaxed phase-matching conditions [26]. SHG intensity, in the plane-wave approximation, is proportional to ${\mathrm{sin}}{{\mathrm{c}}^2}\left( {\Delta k\frac{L}{2}} \right)$, where *L* is the length of the interaction region and $\Delta k = 2{k_\omega } - {k_{2\omega }}$ is the phase mismatch. Here the SHG intensity is maximized in the case of perfect phase-matching ($\Delta k = 0$), and quickly falls off in an oscillatory manor for nonzero $\Delta k$ [32]. Quasi-phase-matching (QPM) is a method of compensating for a nonzero phase mismatch, accomplished through a periodicity of the effective nonlinear susceptibility, ${d_{\mathrm{eff}}}$ [32]. However, as examined by Mertz from an antenna theory framework, even in perfect phase-matching conditions, variations in the medium on the order of the fundamental wavelength give rise to momentum contributions that can result in backward directed SHG emission [33].

In our model, SHG conversion is considered within a single domain, which is a single fibril or close grouping of smaller fibrils, such that each domain has its own associated *Δk*. The overall SHG intensity is the due to contributions from all domains within the focal volume. More forward SHG emission (higher *F*_SHG_/*B*_SHG_ values), are then associated with smaller $\Delta {k_{\mathrm{f}}}$ values and domains on the order of the forward coherence length, ${L_{\mathrm{c,f}}} = \,\frac{{2\pi }}{{\Delta {k_{\mathrm{f}}}}}$, and thus relatively larger fibrils. In contrast, backward emitted SHG is dependent on the axial momentum contributions from the medium, and QPM is more efficient when the interfibrillar spacing is on the order of the backwards coherence length, ${L_{\mathrm{c,b}}} = \,\frac{{2\pi }}{{\Delta {k_{\mathrm{b}}}}}$. Thus, for lower *F*_SHG_/*B*_SHG_ values, the domain size needs to be smaller than ${L_{\mathrm{c,f}}}$ where these are associated with larger *Δk* and smaller fibrils [26].

In addition to probing the SHG creation attributes of conversion efficiency and emission directionality, there is additional richness in making these determinations over a broader wavelength range (∼few hundred nm in the NIR). This is because the SHG response will depend on the domain size relative to the excitation wavelength and potentially will reflect comparative structural determination. For example, we have previously demonstrated that SHG creation metrics exhibit a wavelength dependence in rat-tail tendon [34] and various ovarian tissues [34, 35]. However, the wavelength response has not been well-explained by existing treatments as the underlying physics behind these wavelength dependencies has not been rigorously explored. Specifically, in a simple two-state description the wavelength dependence of the relative conversion efficiency would be primarily expected to arise from ${d_{\mathrm{eff}}}$, the effective second order nonlinear susceptibility, the detuning from resonance, and *Δk*, the phase mismatch arising from the dispersion in the refractive index of the tissue between the fundamental and second harmonic wavelengths. However, our results were not well-fit by this analysis suggesting that the two-state model is either insufficient or incorrect for modeling the wavelength dependence of the SHG conversion efficiency in collagen in the NIR. Additionally, the phase mismatch only varies by about 10% over the wavelength range of the experimental data, and thus cannot account for the wavelength dependence [35]. We therefore must consider size and packing and polarity on the SHG response, both at single wavelengths and across a range of wavelengths.

While our heuristic model can give some insight into the fibril size and spacing without transmission electron microscopy (TEM), it is non-quantitative and can only give relative size scales of the structure with no actual bounds on the physical parameters, including fibril size and packing and also polarity. Then in order to better place bounds on the fibril structure without the need of TEM we need a more complete theory and approach to simulate the SHG emission pattern from various fibril structures found in real tissues. While other treatments have been presented in the literature for SHG from several tissues they have not considered the combination of non-regular 3D structures found in collagenous fibrillar tissues and non-ideal phase-matching conditions [33, 36–46]. Here, we present a new theoretical model to more fully treat the SHG response from type I fibrillar collagen based on QPM theory, of the how the three-dimensional SHG spatial emission pattern is determined by the fibril organization. In addition to the theoretical model, we have developed a computational model for determining the Fourier contributions from the fibril structure, allowing for the examination of the effect of more randomly ordered fibrils on the spatial emission pattern. This also allows for the prediction of the wavelength dependent creation responses.

## 2 Determination of SHG spatial emission and conversion efficiency

Here we summarize the methods we have previously used to extract the spatial distribution of forward and backward components, *F*_SHG_/*B*_SHG,_ and the relative conversion efficiency that served as the basis for developing this more complete computational model [15]. The measured forward and backward components, denoted (*F*) and (*B*), is a coupled response of the emitted *F*_SHG_/*B*_SHG_ (the quantity considered in this study) and subsequent optical scattering of these components governed by the reduced scattering coefficient, *µ*_s_’ at the SHG wavelength. To extract *F*_SHG_/*B*_SHG_, we perform 3 steps. First, we measured the *F*/*B* as a function depth through the tissue, e.g. over 100–200 *µ*m of tissue thickness. Second, we independently measure *µ*_s_’ at the SHG wavelength in a bulk measurement, and third, we perform a forward Monte Carlo simulation to determine *F*_SHG_/*B*_SHG_. Measurements of the relative conversion efficiency are coupled with scattering of the laser fundamental (square of the scattering coefficient *µ*_s_) and *µ*_s_’ at the SHG wavelength. To extract this quantity, we measure the relative intensity of the forward directed signal as a function of depth and then perform a forward Monte Carlo simulation based on the independently measured bulk optical scattering coefficients.

## 3 Theoretical development

To build our generalized QPM model, we begin with the coupled wave equations for SHG, using the slowly varying envelope approximation, as well as assuming a wide beam and negligible depletion of the fundamental electric field as follows:

\begin{equation*}\begin{array}{*{20}{c}} {\frac{{\mathrm{d}{E_{2\omega }}}}{{\mathrm{d}z}} = {\mathrm{i}}\frac{{2\omega }}{{c{n_{2\omega }}}}{\mathrm{d}_{\mathrm{eff}}}{E_\omega }^2\left( {\boldsymbol{r}} \right){\mathrm{e}^{i\Delta kz}}\,} \end{array}.\end{equation*}

To incorporate QPM, ${d_{\mathrm{eff}}}$ is replaced with $d\left( {\boldsymbol{r}} \right)$, allowing the nonlinear susceptibility to vary over the sub-resolution structure of collagen within the focal volume, and then represented as a Fourier series:

\begin{equation*}\begin{array}{*{20}{c}} {\mathrm{d}\left( {\boldsymbol{r}} \right) = {\mathrm{d}_{\mathrm{eff}}}g\left( {\boldsymbol{r}} \right) = {\mathrm{d}_{\mathrm{eff}}}\mathop \sum \limits_{\mathrm{mnl}} {G_{\mathrm{mnl}}}{\mathrm{e}^{i{{\boldsymbol{k}}_{{\mathbf{mnl}}}} \cdot {\boldsymbol{r}}}}\,} \end{array}\end{equation*}

where ${G_{\mathrm{mnl}}}$ are the Fourier components of the normalized structure $g\left( {\boldsymbol{r}} \right)$ and ${{\boldsymbol{k}}_{{\mathbf{mnl}}}}$ are the associated spatial frequencies and also momentum contributions from the medium. In the case in which a single component directly counteracts the phase mismatch, the summation can be reduced to that single Fourier component, neglecting all other terms as they will be negligible in comparison [47]. This is a reasonable assumption for periodic structures, particularly for materials intentionally engineered for efficient QPM. However, as collagen is not a crystalline structure, its inherent randomness will also increase the number of significant Fourier components needed to describe the response. Additionally, it is important to include strong components that do not directly counteract the phase mismatch because any deleterious effects of these components will affect the emission pattern.

For the fundamental field, we use Mertz and Moreaux’s approximation for a Gaussian beam propagating in the *z* direction [33],

\begin{equation*}\begin{array}{*{20}{c}} {{E_\omega }\left( {\boldsymbol{r}} \right) = - i{E_\omega }{\mathrm{e}^{\left( { - \frac{{{x^2} + {y^2}}}{{{w_\rho }^2}}\, - \,\frac{{{z^2}}}{{{w_z}^2}}\, + i\xi {k_\omega }z} \right)}}\,} \end{array}\end{equation*}

where $\xi $ is an empirical value which accounts for the Guoy phase shift, which is the phase shift of *π* that occurs at the focus of a Gaussian beam, and ${w_\rho }$ and ${w_z}$ are the lateral and axial beam waists, respectively, for which we use the following definitions [48]

\begin{equation*}\begin{array}{*{20}{c}} {{w_\rho } = \left\{ {\begin{array}{*{20}{c}} {\frac{{0.320{\lambda _\omega }}}{{\sqrt 2 N{A^{}}}}\,NA \unicode{x2A7D} 0.7} \\ {\frac{{0.325{\lambda _\omega }}}{{\sqrt 2 N{A^{0.91}}}}\,NA &gt; 0.7} \end{array}} \right.\,} \end{array}\end{equation*}

\begin{equation*}\begin{array}{*{20}{c}} {{w_z} = \frac{{0.532{\lambda _\omega }}}{{\sqrt 2 }}\left( {\frac{1}{{{n_\omega } - \sqrt {{n_\omega }^2 - N{A^2}} }}} \right)\,} \end{array}.\end{equation*}

The refractive index is determined through the following empirical formula for collagen in rat-tail tendon [49]:

\begin{equation*}\begin{array}{*{20}{c}} {{n_\omega } = 1.4389 + \frac{{1.588 \times {{10}^4}}}{{{\lambda _\omega }^2}} - \frac{{1.4806 \times {{10}^9}}}{{{\lambda _\omega }^4}} + \,\frac{{4.3917 \times {{10}^{13}}}}{{{\lambda _\omega }^4}}\,} \end{array}.\end{equation*}

When equations (2) and (3) are substituted into equation (1), we now have

\begin{equation*}\begin{array}{*{20}{c}} {\frac{{{E_{2\omega }}}}{{dz}} = i\frac{{2\omega }}{{c{n_{2\omega }}}}{d_{\mathrm{eff}}}{E_\omega }^2\mathop \sum \limits_{\mathrm{mnl}}^{} {G_{\mathrm{mnl}}}{\mathrm{e}^{\left( { - \frac{{{x^2} + {y^2}}}{{{w_\rho }^2}}\, - \,\frac{{{z^2}}}{{{w_z}^2}}} \right)}}{\mathrm{e}^{i{\boldsymbol{\Delta }}{{\boldsymbol{k}}_{{\mathbf{mnl}}}} \cdot {\boldsymbol{r}}}}\,} \end{array}\end{equation*}

in which the momentum contributions and $\xi $ are absorbed into the phase mismatch as ${\boldsymbol{\Delta }}{{\boldsymbol{k}}_{{\mathbf{mnl}}}} = {\boldsymbol{\,}}2\xi {{\boldsymbol{k}}_{\boldsymbol{\omega }}} - {{\boldsymbol{k}}_{2{\boldsymbol{\omega }}}} + {{\boldsymbol{k}}_{{\mathbf{mnl}}}}$.

We then integrate over the focal volume, but since we are operating in the tight focusing limit (where the sample thickness is much greater than the axial beam waist), the limits of integration go to positive and negative infinity

\begin{align*} {E_{2\omega }} = \frac{{i2\omega }}{{c{n_{2\omega }}}}{\mathrm{d}_{\mathrm{eff}}}{E_\omega }^2\mathop \sum \limits_{\mathrm{mnl}} {G_{\mathrm{mnl}}}\mathop \int \limits_{ - \infty }^\infty {\mathrm{e}^{ - \frac{{2{x^2}}}{{{w_\rho }^2}}}}{\mathrm{e}^{i\Delta {k_{\mathrm{mnl},x}}x}}\mathrm{d}x\mathop \int \limits_{ - \infty }^\infty {\mathrm{e}^{ - \frac{{2{y^2}}}{{{w_\rho }^2}}}}{\mathrm{e}^{i\Delta {k_{\mathrm{mnl},y}}y}}\mathrm{d}y\mathop \int \limits_{ - \infty }^\infty {\mathrm{e}^{ - \frac{{2{z^2}}}{{{w_z}^2}}}}{\mathrm{e}^{i\Delta {k_{\mathrm{mnl},z}}z}}\mathrm{d}z\,.\end{align*}

Upon integration we get the following result for the second harmonic electric field:

\begin{equation*}\begin{array}{*{20}{c}} {{E_{2\omega }} = i{{\left( {\frac{\pi }{2}} \right)}^{{\raise0.7ex\hbox{$ \scriptscriptstyle 3$} \!\mathord{ \scriptscriptstyle / {\vphantom {3 2}}} \!\lower0.7ex\hbox{$ \scriptscriptstyle 2$}}}}\frac{{2\omega {w_\rho }^2{w_z}}}{{c{n_{2\omega }}}}{d_{\mathrm{eff}}}{E_\omega }^2\mathop \sum \limits_{\mathrm{mnl}}^{} {G_{\mathrm{mnl}}}{\mathrm{e}^{ - \frac{{\Delta {k_{\mathrm{mnl},x}}^2{w_\rho }^2}}{8}}}{\mathrm{e}^{ - \frac{{\Delta {k_{\mathrm{mnl},y}}^2{w_\rho }^2}}{8}}}{\mathrm{e}^{ - \frac{{\Delta {k_{\mathrm{mnl},z}}^2{w_z}^2}}{8}}}\,} \end{array}.\end{equation*}

This result is very similar in form to that of Mertz [33] even though their model was based on antenna theory and ours is based on QPM, and when solved in 1D, provide exactly matching results for the simple sinusoidal example given in their work. We have extended and generalized this to higher dimensions, as seen in equation (9), and we will apply it here to non-periodic structures, corresponding to more realistic collagen architecture in tissues.

## 4 Computational model

For periodic or lattice structures, ${G_{\mathrm{mnl}}}$ and ${{\boldsymbol{k}}_{{\mathbf{mnl}}}}$ can be determined analytically, however collagen in tissue is not a crystalline structure and thus we need a computational model.

**Figure 1.**
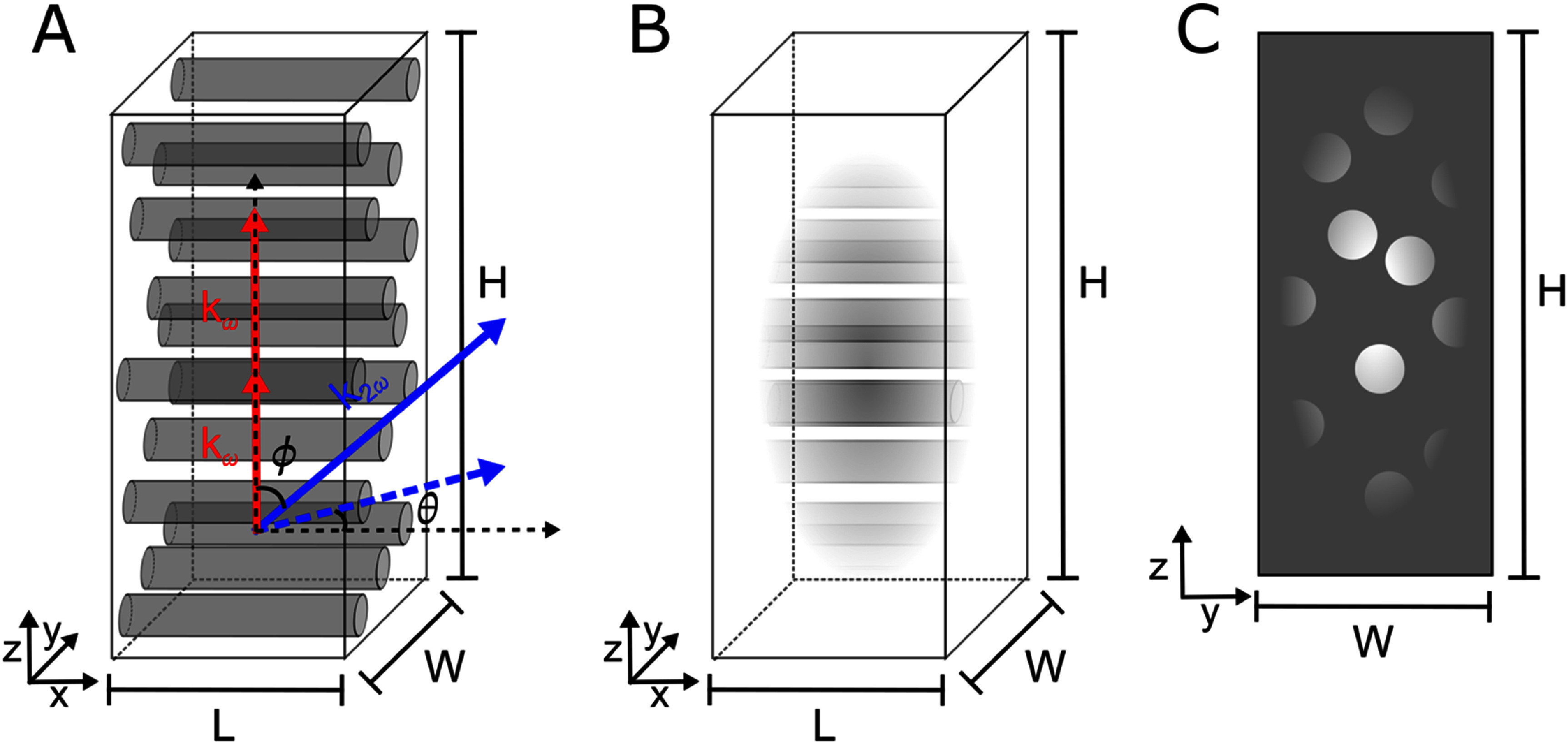
(A) Cartoon depiction of the computational model of collagen fibril structure. Fibrils are modeled as cylinders oriented orthogonally to the fundamental wave vector, ${k_\omega }$ (red arrows), in a non-interacting background of size *L* by *W* by *H*, where *L* = *W* = 1.4 *µ*m and *H* = 6 *µ*m. The resulting second harmonic wave vector, ${k_{2\omega }}$, is depicted as a blue arrow at inclination angle $\phi $ with respect to ${k_\omega }$, and azimuthal angle $\theta $, where the dashed blue arrow is the projection of ${k_{2\omega }}$ onto the *x*–*y* plane. (B) Depicts the model structure with the Gaussian filter matching the laser parameters that represents focal volume applied. (C) Is the 2D *y*–*z* slice (from the center in *x*) of the structure and is how the models are represented in the results for clarity.

A cartoon of our model is depicted in figure 1. We represent collagen fibrils as cylinders in a non-interacting background of length *L*, width *W*, and height *H*, where *L* and *W* are four times the maximum lateral beam waist and *H* four times the maximum axial beam waist for the wavelength range used in computation. In order to prevent edge effects, we want features in the edges of the volume to be zero or very close to zero. This is achieved by applying a Gaussian filter that matches laser parameters for each wavelength to the model structure to represent as the focal volume. Four times the beam waist was chosen to balance the intent to be very close to zero at the edges with computation power and precision concerns with the size of a single voxel (these models are 100 by 100 by 300 voxels for determining the Fourier components). The momentum contributions are determined as follows:

\begin{equation*}\begin{array}{*{20}{c}} {\left\{ {\begin{array}{*{20}{c}} {{k_{mn{\mathrm{l}},x}} = 2\pi m/L} \\ {{k_{mn{\mathrm{l}},y}} = 2\pi n/W} \\ {{k_{mnl,z}} = 2\pi l/H} \end{array}\,} \right.} \end{array}\end{equation*}

where *m, n*, and *l* are integers ranging from negative half the voxel length to positive half the voxel length in each dimension.

The Fourier components ${G_{\mathrm{mnl}}}$ are calculated via the discontinuous fast Fourier transform (DFFT) [50]. Here, a discontinuous function is split piecewise into smooth functions such that the Fourier transform can be defined as a summation of the Fourier transforms of the individual continuous regions. This is then evaluated through a double interpolation method, where each Fourier transform is estimated through Gaussian–Legendre quadrature, and the functions at each Gaussian–Legendre node is obtained through Lagrange interpolation [50]. For our specific implementation of the DFFT, we chose to directly compute the exponentials at the Gaussian–Legendre nodes rather than perform a second Lagrange interpolation. The DFFT and our implementation of it is described in more detail in the supplement. Our Gaussian–Legendre quadrature is of order 128 (*q* = 128) and as our functions (i.e. the fibrils) are effectively linear as they are Gaussian over small regions, our Lagrange interpolations are of order 2 (*p* = 2). The Gaussian–Legendre nodes and weights are calculated once using the symbolic toolbox in MATLAB, which are then stored along with the Lagrange interpolation factors that do not depend on the specific function, which are also computed once, for use in each model. We then apply the DFFT method along each dimension of the model to obtain the Fourier components. This is implemented in MATLAB and evaluated using the high throughput computing (HTC) cluster at UW-Madison’s Center for High Throuput Computing (CHTC).

Then, to determine the spatial emission pattern, we allow the second harmonic wave vector to point in an arbitrary direction as defined by angles *θ* and *φ* (as depicted in figure 1). As we have already assumed the fundamental wave propagates along the *z*-axis, we can define the phase mismatch in each direction as follows:

\begin{equation*}\begin{array}{*{20}{c}} {\left\{ {\begin{array}{*{20}{c}} {\Delta {k_z} = 2\xi {k_\omega } - {k_{2\omega }}\cos \phi } \\ {\Delta {k_{x\,}} = - \,{k_{2\omega }}\cos \theta \sin \phi } \\ {\Delta {k_{y\,}} = - \,{k_{2\omega }}\sin \theta \sin \phi } \end{array}} \right.\,} \end{array}.\end{equation*}

Then, we let *θ* be of the range -π to π and *φ* be of the range 0 to *π*, with 200 points for each, and then evaluate equation (9) at each combination of points, including the Fourier components previously calculated. The resulting second harmonic electric field is then multiplied by its complex conjugate as this is proportional to the intensity, and then plotted in spherical coordinates for the emission pattern.

In order to calculate the *F*_SHG_/*B*_SHG_ ratio from the spatial emission pattern, we first divide the pattern into forward and backward emission, and then separate out the individual lobes by finding the local minima along the *x*–*z* plane, as the majority of the lobes lie on this plane. We then select all the points associated with an individual lobe and use the MATLAB function boundary to calculate the volume of the lobe. This function creates a triangulation representing the boundary of the specified points and also returns the volume of that boundary. The separation of lobes step is necessary to prevent the boundary function from connecting the lobes into a single structure. We next sum over each of the lobes in the forward and backwards direction and divide the total forward volume by the total backwards volume to obtain the *F*_SHG_/*B*_SHG_ for that pattern. For the integrated intensities, we sum the total volume of all the lobes. This calculation is described in more detail in the supplement.

## 5 Results

### 5.1 Validation of computational model

**Figure 2.**
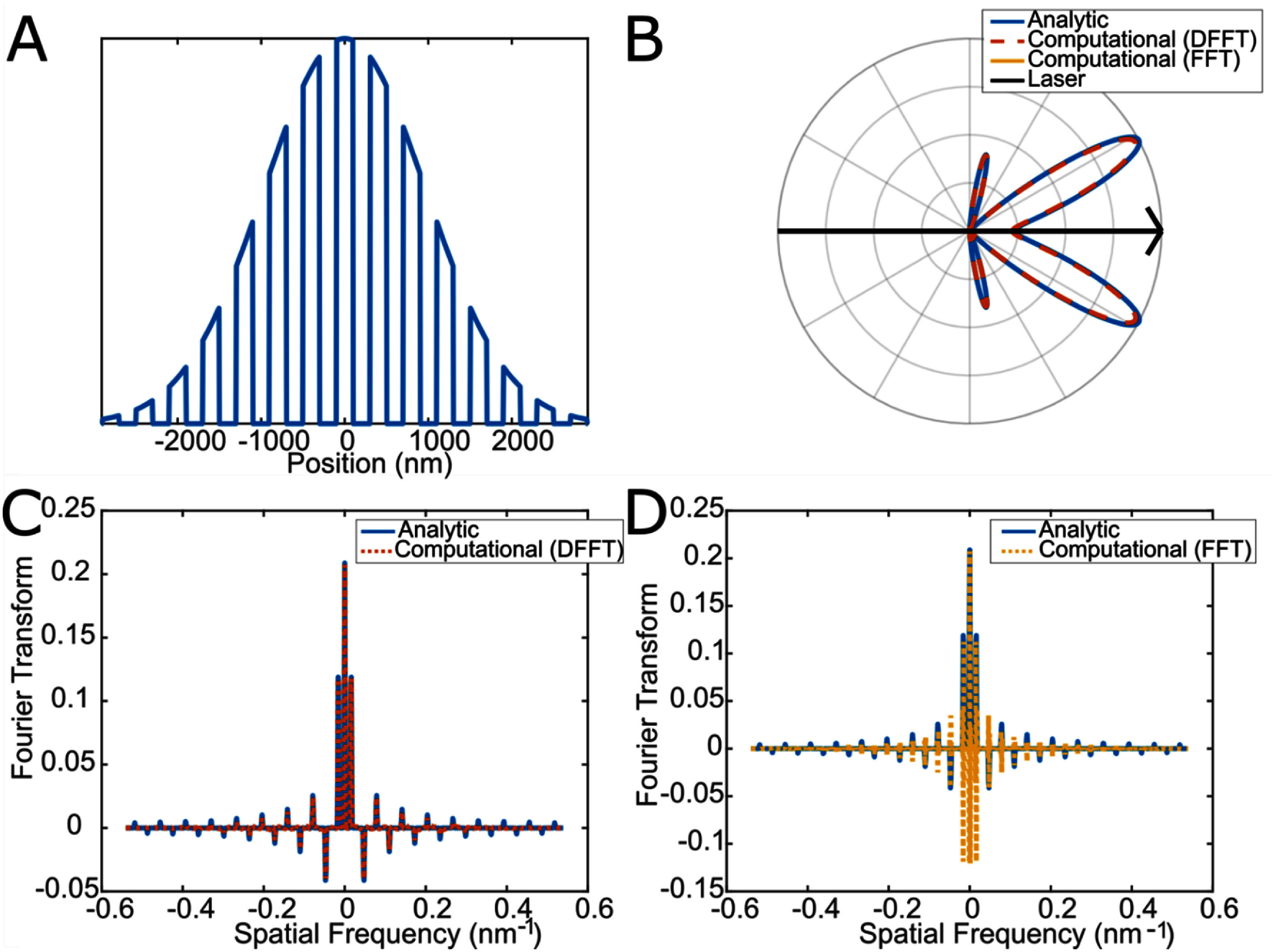
Validation of computational model in 1D. (A) Periodic model structure with Gaussian filter matching laser parameters applied. (B) Polar plot of the spatial emission of SHG intensity for the analytic calculation of ${G_{\mathrm{mnl}}}$ in solid blue, computational calculation of ${G_{\mathrm{mnl}}}$ via DFFT in dashed red, and via FFT in yellow (not visible as it is multiple orders of magnitude smaller in peak intensity). The black arrow represents the laser propagation direction. There is a 2.12% error in peak lobe intensity between the analytic FT and DFFT. (C) and (D) are the real parts of the Fourier components comparing the analytic (solid blue) to the DFFT (dotted red) and FFT (dotted yellow), respectfully.

To validate our computational model we used periodic structures, as ${G_{\mathrm{mnl}}}$ and ${{\boldsymbol{k}}_{{\mathbf{mnl}}}}$ can be determined analytically in these cases. First, for the 1D case, the structure used a square wave centered around zero, with 200 nm pulse widths and a period of 400 nm, as depicted in figure 2(A). The overall length of the structure is 6 times the axial beam width for a wavelength of 800 nm and a Gaussian filter is applied for this wavelength. Figure 2(B) depicts the resulting emission pattern, in which the DFFT computational model accurately matches the analytic solution (2.12% error in peak lobe intensity), while the FFT emission pattern is not visible as it is 3 orders of magnitude smaller than the analytic solution. The reason for this disparity between the DFFT and FFT models can be seen in figures 2(C) and (D), which compares the Fourier transforms (${G_m}\,$ vs ${k_m}$) for the analytic and DFFT (C) or FFT (D). The DFFT matches the analytic FT quite well for the lower order peaks, while the FFT has strong negative peaks associated with all of the peaks, even the lower order terms. This confirms that the DFFT is a better Fourier transform algorithm than the FFT for this purpose.

**Figure 3.**
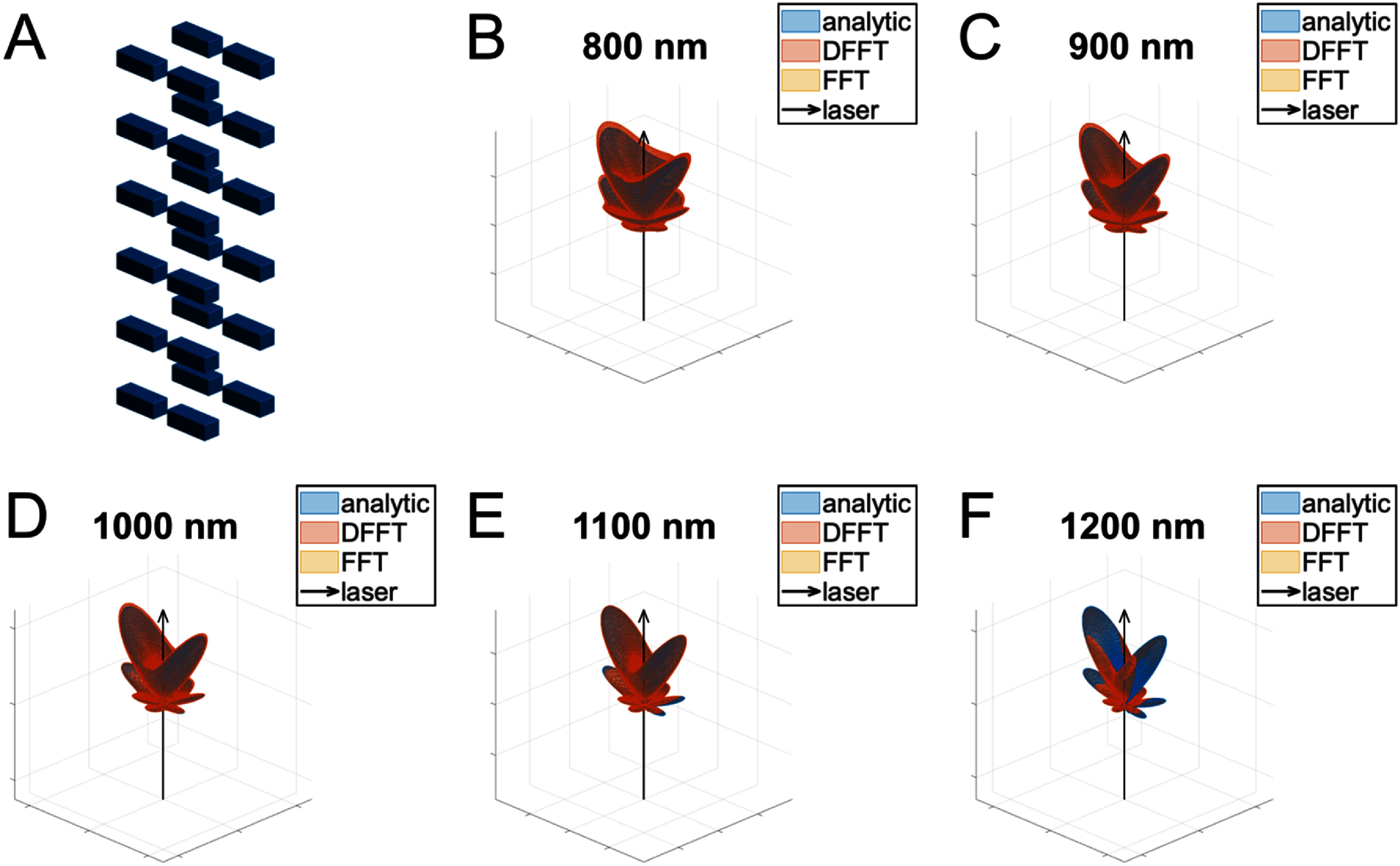
Validation of computational model in 3D. (A) Periodic structure of rectangular prisms in a rectangular lattice (Gaussian filter not included in depiction for clarity). (B)–(F) Comparison of analytic (blue) to computational (red for DFFT, yellow for FFT) SHG spatial emission for fundamental wavelength of 800–1200 nm. Laser propagation direction shown as a black arrow.

Next, for our validation in three dimensions, we use a rectangular prism motif in a rectangular lattice, as depicted in figure 3(A). The rectangular prisms were chosen instead of cylinders for a more reasonable computation time for the analytic solution (7–10 d for the full wavelength range). The resulting emission patterns can be seen in figures 3(B)–(F). While the percent error in peak lobe intensity varies between 0.73% for 1100 nm and 6.18% for 800 nm, the overall shape of the computational model matches very well that of the analytic model. This is deemed sufficient as we are more concerned with the emission profile than the absolute intensity. Additionally, when the structure parameters are slightly mismatched between the computational and analytic models (a difference in rectangular prism of height of 10% and the locations of the centers of the rectangular prisms varying by up to 20 nm), the emission patterns were substantially different from one another (not shown).

### 5.2 Role of fibril polarity

**Figure 4.**
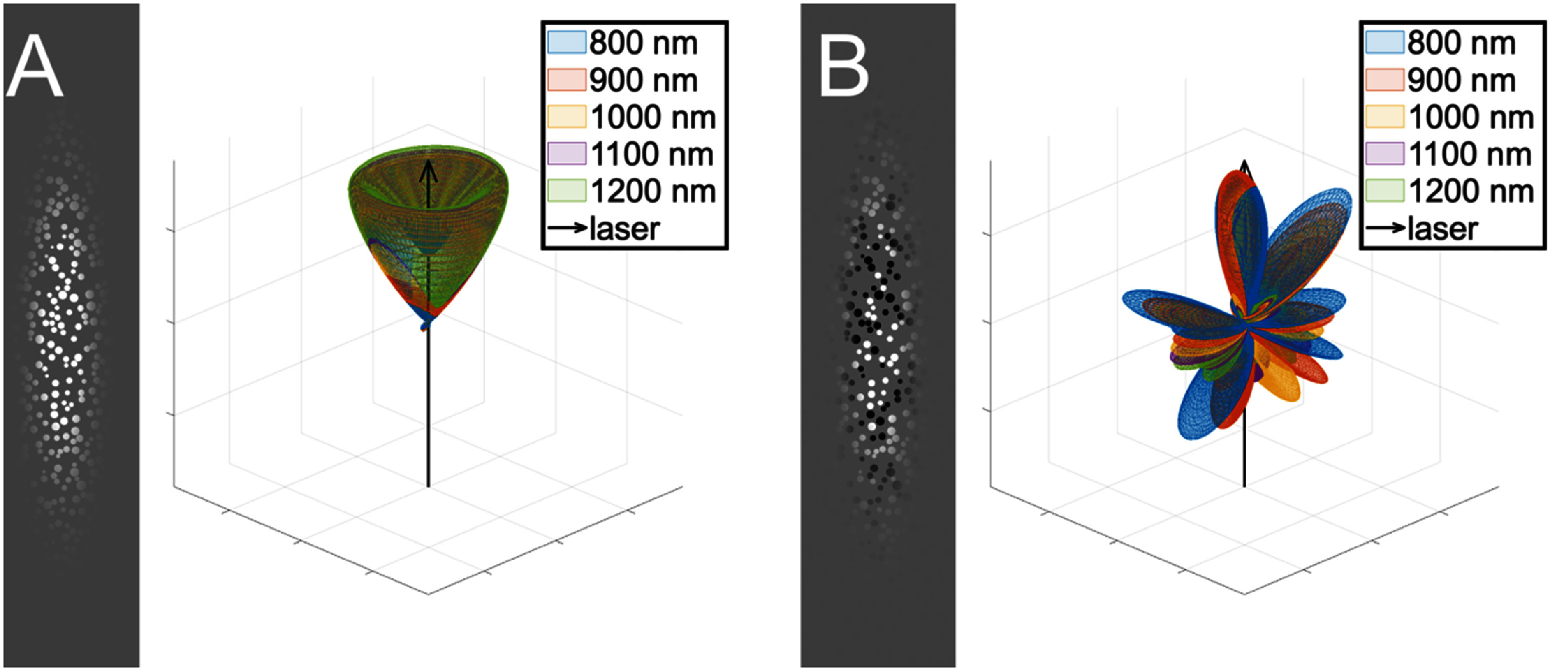
Comparison of aligned versus random fibril polarity. For (A), aligned fibrils, and (B), fibrils with random polarity, on the left is a 2D slice of the fibril structure with the Gaussian filter applied, and on the right is the SHG emission pattern over the wavelength range of 800–1200 nm, with the black arrow depicting the laser propagation direction.

Electron microscopy of longitudinally oriented collagen fibrils in rat-tail tendon has shown that the fibril polarity is random [51]. To investigate the role of polarity in the SHG creation, we compare the emission patterns of aligned and random fibril polarity. Fibril polarity is represented by assigning each fibril a value of ±1 for its normalized ${d_{\mathrm{eff}}}$. When all fibrils have the same polarity (either all +1 or −1), regardless of other structure parameters, the emission pattern is highly forward directed. An example of this is shown in figure 4(A). However, this highly forward emission is inconsistent with experimentally observed F_SHG_/B_SHG_ ratios in real tissues, which typically are on the order of 1–10 [26]. On the other hand, when the fibril polarity is randomly assigned ±1, as in figure 4(B), the emission pattern is much more varied. This suggests that collagen fibrils do not have aligned polarity within fibers in tissues, which is consistent with electron microscopy data of rat-tail tendon in the literature [51]. Although the highly forward directed pattern of the aligned fibrils does not match typical *F*_SHG_/*B*_SHG_ values for collagen, the cone structure is very similar to the results from Mertz as well as the work from Rouède *et al* for myosin in muscle, which does have aligned polarity sub-resolution structures [33, 43].

### 5.3 Parameter sweep and bounds on fibril size and packing from emission directionality

**Figure 5.**
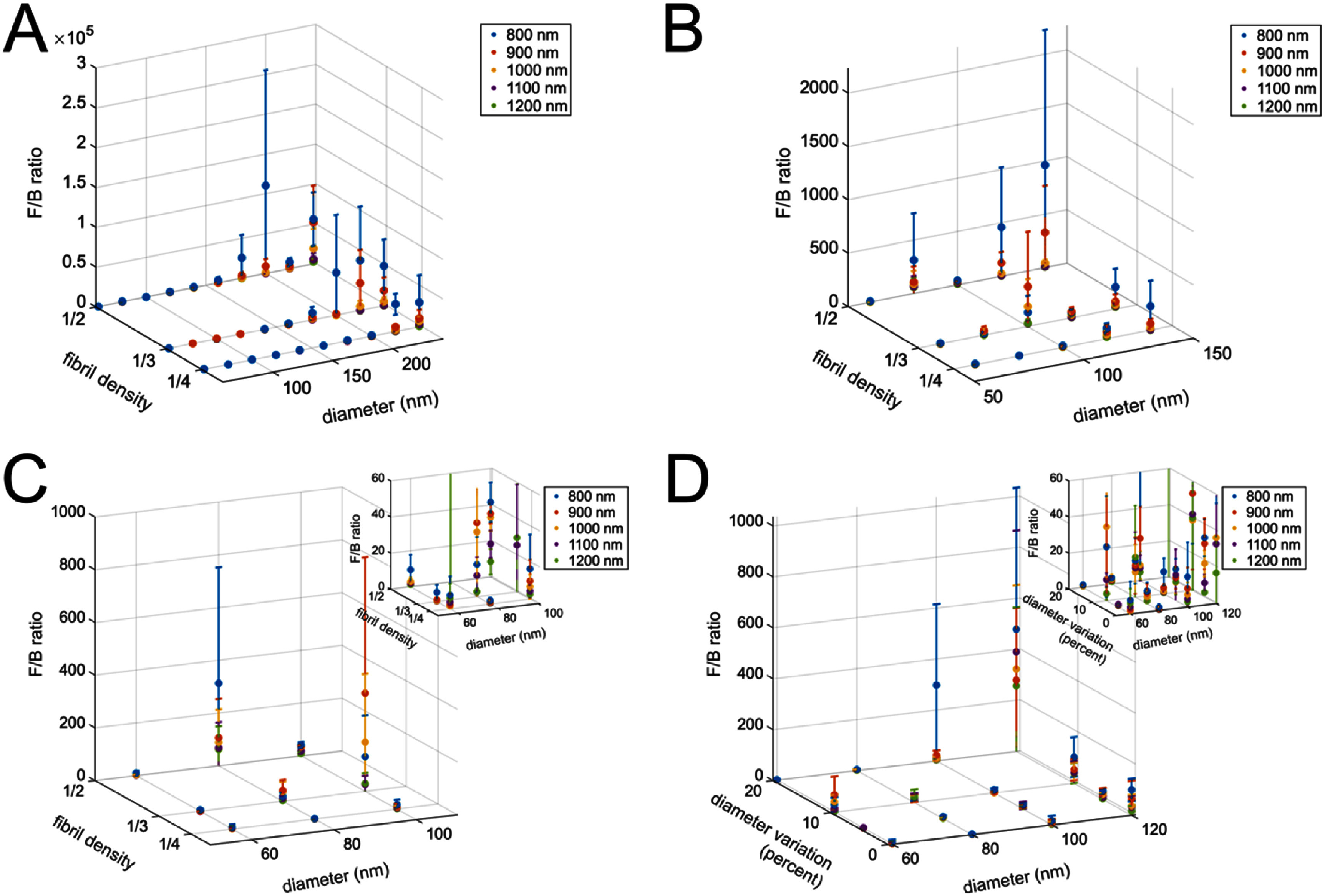
Forward vs backwards SHG emission over parameter space. F_SHG_/B_SHG_ ratios for fibril densities of ¼–½ the focal volume and fibril diameters of (A) 60–240 nm, (B) 60–140 nm, and (C) 60–100 nm, over a wavelength range of 800–1200 nm. (D) F_SHG_/B_SHG_ ratios at a fibril density of ¼ for mean fibril diameters of 60–120 nm with diameter variation of 0%–20% over the same wavelength range. The inserts in (C) and (D) are truncated in F_SHG_/B_SHG_ at 60 for clarity in a more physical F_SHG_/B_SHG_ range. There are three models for each parameter set across all plots.

In order to find bounds on the fibril structure parameter space, we need to compute *F*_SHG_/*B*_SHG_ values from the emission patterns over a parameter space for which we have prior experimental or simulation data. Specifically, we use the wavelength range of 800–1200 nm [27, 34, 35]. Fibril diameters are set from 60 to 240 nm, which generally corresponds to fibril diameters as measured through electron microscopy [27, 51–53]. We represent a range in fibril packing densities as the fibrils filling ¼–½ of the focal volume. Higher packing densities were not initially explored due to constraints on computation time in generating the fibril structures. Moreover, there is interstitial fluid separating fibrils within fibers, limiting the packing density in tissues. For each parameter set, three individual models were computed with randomly generated fibril placement and polarity. Figure 5(A) shows the full parameter space explored. From this we can see that very large fibril sizes results in highly forward emission, with the *F*_SHG_/*B*_SHG_ values three or more orders of magnitude larger than what is observed experimentally [26, 27, 54].

For all fibril densities, the *F*_SHG_/*B*_SHG_ ratios become outside the experimental range for fibril diameters above 120 nm. While this seems to be inconsistent with electron microscopy data [51–53], as the size distribution of tendon fibril diameters can extend to 200 nm (although the average is smaller), this may be explained through measurement precision limitations. *F*_SHG_/*B*_SHG_ ratios in tendon are on the order of 10, which is at the edge of the dynamic range of collection instrumentation (∼15–20 fold), and thus the measured values may be lower than the actual values. Figure 5(B) zooms in along the diameter axis of the parameter space with fibril diameters of 140 nm and below. At this zoom we can more clearly see that *F*_SHG_/*B*_SHG_ values generally increase with increasing fibril density. Figure 5(C) further zooms in to under 100 nm diameters, with the inset plot truncating the *F*/*B* ratio at 60 to see more detail in the experimentally observed range.

For the second parameter sweep we kept the fibril density constant at ¼, as this gave the most similarity with experimentally observed *F*_SHG_/*B*_SHG_ values in the previous parameter sweep, and the mean fibril diameter was allowed to vary between 60 nm and 120 nm. We now, however, investigate variation in fibril diameter size. The fibril diameters were randomly selected from a normal distribution about the mean diameter with a standard deviation from 0% to 20% of the mean diameter. The resulting *F*_SHG_/*B*_SHG_ are plotted in figure 5(D). Interestingly, these values generally increase with increasing variation in fibril diameter, suggesting that the larger fibrils in the distribution have a greater effect on how forward directed the emission is than the smaller fibrils in the distribution.

It is important to note that that the experimental *F*_SHG_/*B*_SHG_ values were determined by averaging over full fields of view (∼170 × 170 *µ*ms) and likely did not account for heterogeneity. Notably, at the 890 nm excitation wavelength we examined the distribution in *F*_SHG_/*B*_SHG_ in ovarian tissues where more varied fibers had a broad range of *F*_SHG_/*B*_SHG_ (3–15 within the same field of view) [55]. Thus, these computational results and our prior work showed the importance of the actual distribution on the spatial emission pattern. Moreover, this allows us to make estimates of sizes in the tissue and their respective contributions.

We next compare our computational results of the wavelength dependent *F*_SHG_/*B*_SHG_ with those of our prior experimental work on ovarian cancer and tendon over the same wavelength range (800–1200 nm) [27, 35]. We had found that the *F*_SHG_/*B*_SHG_ from tendon increased slightly from 6.8/1–8.5/1 with increasing wavelength. These values for ovarian tissues were either wavelength independent or increased slightly at longer wavelengths. We had argued that these values increased due to decreased dispersion in refractive index between the fundamental wavelength and *λ*_SHG_ and then having longer coherence lengths. While dispersion was included in our computational model, we found here that the *F*_SHG_/*B*_SHG_ values generally decreased with increasing wavelength but were more varied when closely examining size and packing. Specifically lower *F*_SHG_/*B*_SHG_ values and decreased wavelength dependence are associated with smaller fibrils, while larger values and a stronger wavelength dependence are associated with larger fibrils or tightly packed smaller fibrils [26, 27].

We can use these general results to place bounds on the fibril parameters in tendon and ovarian tissues. In figures 5(A)–(C) we demonstrated that increasing fibril size results in more forward directed SHG emission. In figure 5 we identified regimes of fibril size or higher packing densities where *F*_SHG_/*B*_SHG_ values increase with increasing wavelength, where for example this is exemplified by 1/3 packing density and fibril diameters of 80 and 100 nm. Using these computational results, we determined that our previously extracted *F*_SHG_/*B*_SHG_ values would arise from fibril diameters under 120 nm. Notably, these values are consistent data from mouse tendon (∼100–150 nm) and our ovarian TEM of 50–100 nm [27]. These comparisons provide evidence that our computational model in conjunction with our prior experimental studies of the wavelength dependent *F*_SHG_/*B*_SHG_ predicts physically reasonable fibril size and packing.

### 5.4 Wavelength dependence of SHG conversion efficiency

**Figure 6.**
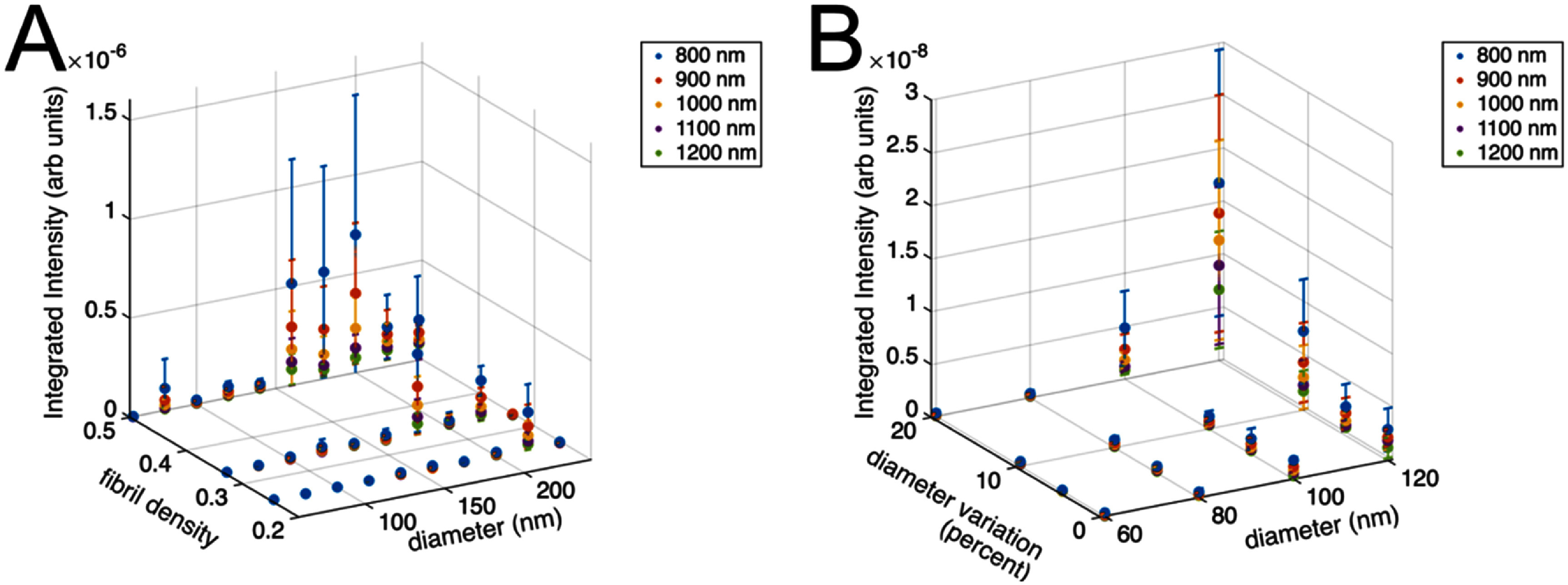
Integrated SHG intensity over parameter space. (A) SHG intensities for fibril densities of ¼–½ the focal volume and fibril diameters of 60–240 nm over a wavelength range of 800–1200 nm. (B) SHG intensities at a fibril density of ¼ for mean fibril diameters of 60–120 nm with diameter variation of 0%–20% over the same wavelength range. There are three models for each parameter set across all plots.

Additionally, our computational framework allows us to probe the SHG conversion efficiency, represented through the integrated intensity, as a function of fibril size and packing. For each parameter sweep we also calculated the integrated SHG intensities (conversion efficiency) from the emission patterns. The wavelength dependence of the integrated intensity for this parameter set is shown in figures 6(A) and (B). The integrated intensity is determined by adding the total forward and backward intensity volumes instead of simply dividing for the *F*_SHG_/*B*_SHG_. Here we find that in addition to becoming more forward-directed, the overall intensity increases for larger fibrils, denser fibril packing, and increased diameter variation. Additionally, the wavelength dependence of individual parameter sets is more consistent in intensity, with intensity decreasing with wavelength, while there is more variation in wavelength dependence for the emission directionality, suggesting the exact morphology is more important in determining the latter response.

We can compare these predictions to our prior work, where we determined the wavelength dependence of SHG conversion efficiency for mouse tail tendon over the range of 800–1200 nm. While experimentally we found a decrease at longer wavelengths, the results did not match well with that predicted by the two-state model of the effective nonlinear susceptibility [35]. Our computational model predicts a similar decrease in SHG conversion with increasing wavelength dependence where this overall decrease was consistent across the fibril structure parameter space (figure 6). However, we did find that larger fibrils contribute more to the response to be consistent with 3-fold change in intensity of our prior work. One should note our computational result here assumes a constant ${d_{\mathrm{eff}}}$ with wavelength, which is a reasonable approximation due to the small change in refractive index over the wavelength range, and yet there is still a large wavelength dependence.

### 5.5 Role of spatial heterogeneity and polarity clustering

Based on the results in sections 5.3 and 5.4, we have found that the heterogeneity in the fibril structure is important in determining the SHG creation attributes. In order to further explore the effects of heterogeneity in the fibril structure on the SHG spatial emission pattern and resulting *F*_SHG_/*B*_SHG_ values, we first investigate the variation within a single randomly generated parameter set. Then to separate out the effects of different fibril structure features on the emission pattern, we compare lattice structures to random structures, both in fibril placement and in fibril polarity.

**Figure 7.**
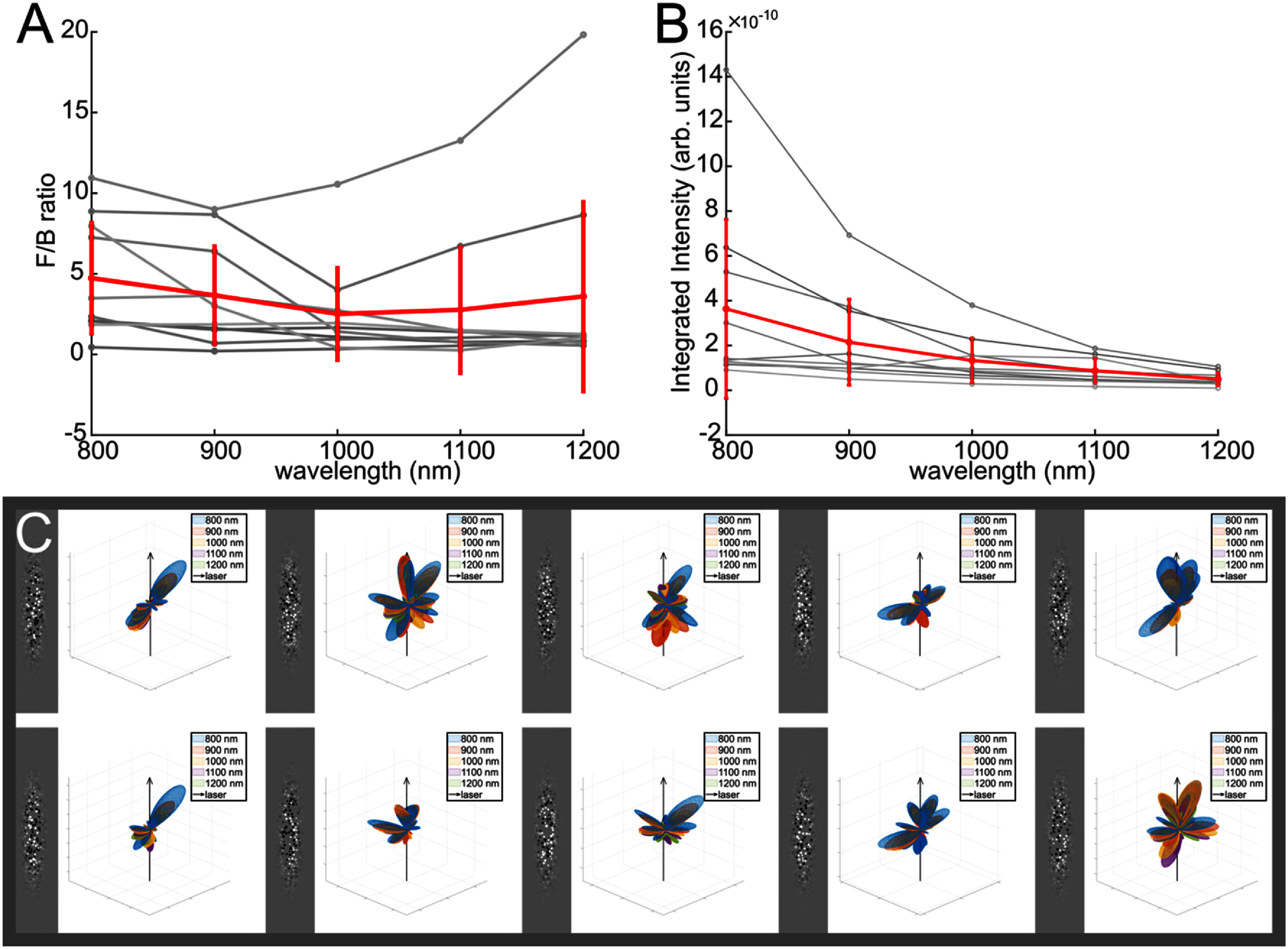
Heterogeneity in a single parameter set (10 total models, 60 nm diameter fibrils with 20% diameter variation, with a fibril density of ¼). For both (A), F/B ratio versus wavelength, and (B), integrated intensity versus wavelength, the individual models are in grey and the mean is in red. (C) 2D fibril structure slices and resulting SHG emission patterns over the wavelength range of 800–1200 nm for each of the individual models, showing a high variability in emission pattern for the same parameters. The black arrows represent the laser propagation direction.

In the parameter sweep, many of the error bars for the *F*_SHG_/*B*_SHG_ values of single parameter sets are quite large, so we investigated the cause of this variability by computing the emission patterns and resulting ratios for 10 models of a single parameter set (60 nm diameter fibrils with 20% diameter variation, with a fibril density of ¼). Figure 7(A) shows the variation in *F*_SHG_/*B*_SHG_ over the different models in a single parameter set. The emission patterns for each individual model are shown in figure 7(C). From this we find that there is great diversity in the emission patterns, which accounts for the variation in *F*_SHG_/*B*_SHG_, despite all models being generated from the same set of parameters. Figure 7(B) shows the corresponding results for the relative conversion efficiency. While there is some heterogeneity in the SHG conversion efficiency, these results are less affected by the heterogeneity than those for the emission directionality.

**Figure 8.**
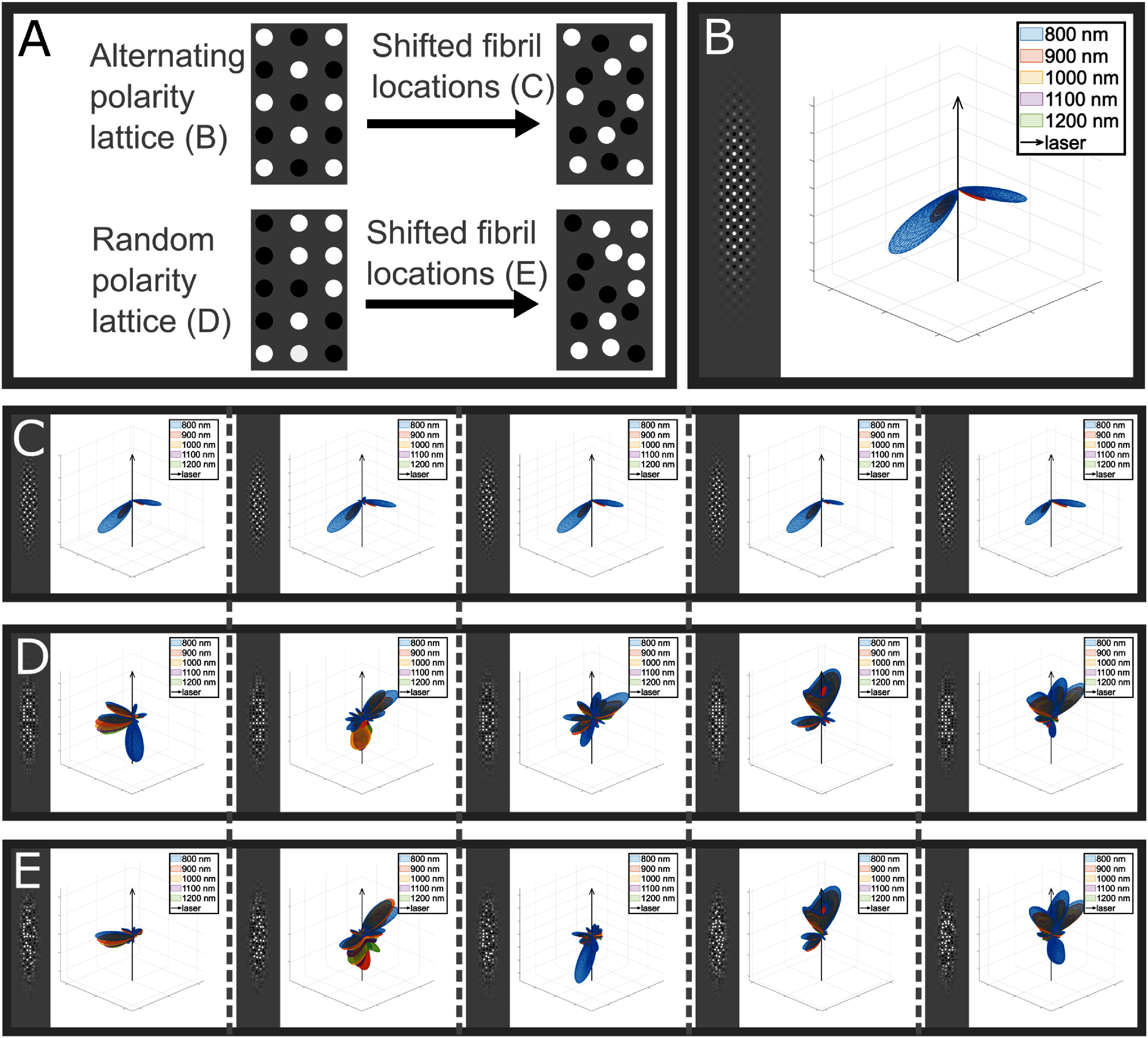
Separating fibril location from polarity clustering. (A) Cartoon depicting testing conditions for (B)–(E). 2D slice of the fibril structure and resulting emission patterns over the wavelength range of 800–1200 nm, with the black arrow representing the direction of laser propagation for (B) alternating polarity fibrils arranged in a rectangular lattice, and five examples each for (C) alternating polarity with shifted fibril locations, (D) random polarity fibrils in a rectangular lattice, and (E) random polarity with shifted fibril locations. Vertically across (C)–(E), there are the same fibril location shifts for (C) and (E), and the same polarity placements for (D) and (E).

Since these models were generated with both random fibril locations and random fibril polarity, of which the latter can produce domains of clustering of aligned fibrils, it is unclear which has a greater effect on the emission pattern. In order to tease apart the effect of fibril location vs clustering of fibril polarity on the emission pattern, we start with a square lattice, as this allows for a consistent fibril placement. The fibrils are all 60 nm in diameter and the period of the lattice was chosen such that the fibril density was close to ¼ as possible. To test the effect of the clustering of fibril polarity, we compare alternating polarity in the lattice (no aligned fibril domains) to random fibril polarity in the lattice. We then can shift these lattices into more random structures by allowing each of the fibrils to fluctuate in location, letting us probe the effect of fibril placement on the emission pattern. A cartoon depiction of this comparison can be seen in figure 8(A). Figure 8(B) depicts the emission pattern for the alternating polarity lattice structure along with the structure itself. This pattern consists of two backwards lobes very close to the lateral plane. When we compare this to the emission patterns of alternating polarity structures with shifted fibril locations (figure 8(C)), we can see that there are some alterations of the emission pattern from the alternating lattice structure, but still very similar to the pattern in figure 8(B). However, the emission patterns for the random polarity lattices, which have domains of aligned fibrils (figure 8(D)), are highly varied and do not appear similar to the alternating lattice emission pattern. Additionally, when we further allow these random polarity lattices to fluctuate in fibril locations (figure 8(E)), the resulting emission patterns do vary from the lattice structures, but there is far more variation across the random polarity structures. This suggests that the clustering of fibril polarity such that there are domains of aligned fibrils has a far greater effect on the SHG emission pattern than the exact locations of the fibrils.

## 6 Discussion

Through the combination of experiments and modeling, we have previously used the spatial emission directionality or created forward-backward ratio, *F*_SHG_/*B*_SHG_, to characterize trends in the sub-resolution collagen fibril size and packing in numerous tissues. Our overarching premise has been that normal and diseased tissues (for example cancers, fibroses, and connective tissue disorders) will have different fibril architectures on the size scale of *λ*_SHG,_ where, more specifically, diseased states will often have a less ordered structure due to collagen remodeling and thus have a shorter coherence length [15, 26, 27, 56]. We have further used this approach to study the wavelength dependence of *F*_SHG_/*B*_SHG_ emission directionality in several tissues [27, 35]. This method provides additional richness as it provides insight into the distribution of fibril sizes relative to the laser excitation and SHG wavelengths. While this heuristic model has proven very useful in this regard, it is non-quantitative and has several simplifying assumptions. Here, we expanded on the ideas in that model to develop a more rigorous theoretical and computational model based on QPM to predict the spatial emission pattern of SHG from the fibril size, packing and polarity structure in collagen. As described below, our new treatment addresses these limitations at both single wavelengths and also the wavelength dependence therein. We also provide comparisons to previous treatments by others to show the greater generality of our model and better applicability to realistic tissue structure. We note that in our treatment, the phase mismatch refers to the difference between the fundamental (or input) wave and the second harmonic wave and is not related to the phase difference in the SHG signal probed by interferometric methods [57].

Though QPM has been used previously to describe the phase-matching in collagen and other biological tissues and for theoretically determining the spatial emission pattern thereof [37–39, 41–43], many of these treatments use several simplifying approximations. These have included employing two dimensional lattice structures and single valued Fourier components [37–39]. Only in Rouède *et al*’s work on myosin in muscle were more than a single Fourier component considered, where they assumed a crystalline lattice. However, that is more appropriate in myosin due to its regular packed hexagonal structure. In contrast, collagen has a much more complex fibrillar architecture [41–43]. Specifically, the harmonophore in myosin is the myofibril which has a diameter about 1–2 *µ*ms. These are closely hexagonally spaced with high regularity into a crystal-like structure. The myofibrils are composed of regularly packed thick filaments (and other non-SHG active components such as actin) where the size and spacing is highly conserved. In stark contrast, the collagen fibers seen in SHG microscopy have an underlying structure of fibrils that crosslink into fibers. The diameters and spacing of fibrils are varied even in highly organized tissues such as tendon, where for example, the size range is ∼50–100 nm with irregular packing. Thus, these computational based structures are not reflective of the collagen fibril architecture in tissues. While not QPM based, Mertz utilized a Fourier treatment of the structure with multiple Fourier components [33, 36]. Though their work was largely instrumental in establishing a theoretical basis for backwards emitted SHG, it assumed perfect phase-matching, which does not occur in collagenous tissues, and also did not consider more realistic fibrillar structures. Outside of QPM approaches, there have been a few other theoretical approaches to SHG spatial emission in fibrillar collagen, some of which did not include effects of the fibril structure [40], while others allowed for random fibril placement [44–46]. However, none of these have included phase-matching considerations. By contrast to the previous literature, our work is three dimensional, incorporates the phase mismatch, and considers many Fourier components to allow for the modeling of random fibril structures and can be used to calculate the spatial emission from biomimetic structures. In table 1 we summarize the attributes of the theoretical approaches used in these prior reports as well as the more generalized aspects included in our model.

**Table 1.** Summary of considered attributes of theoretical approaches of SHG emission direction from prior reports.

	3D	$\Delta k \ne 0$	Multiple Fourier components	Random structures
Tian *et al* [37]		*X*		
Tian *et al* [38]		*X*		
Shen *et al* [39]		*X*		
*Rouède *et al* [41]	*X*	*X*	*X*	
*Rouède *et al* [42]	*X*	*X*	*X*	
*Rouède *et al* [43]	*X*	*X*	*X*	
Mertz and Moreaux [33]	*X*		*X*	
Moreaux *et al* [36]	*X*		*X*	
Ranasinghesagara *et al* [40]	*X*		N/A	
Brown *et al* [44]	*X*		N/A	*X*
Rivard *et al* [45]	*X*		N/A	*X*
van der Kolk *et al* [46]	*X*		N/A	*X*
This work	*X*	*X*	*X*	*X*

* indicates work done on myosin rather than collagen.

We next discuss our predictions of the spatial emission pattern and also the relative SHG conversion efficiency by hypothesis testing the roles of fibril size, spacing and polarity. By including many Fourier components determined via computational modeling (equation (9)) we can incorporate the inherent randomness in the fibril structure of collagen, which would not be possible through analytic calculations that require assumptions of a lattice arrangement of fibrils. We found that by incorporating this randomness, even with a single parameter set (size and spacing of fibrils), there was a high degree of heterogeneity between SHG emission patterns across individual models, thus showing that the spatial emission pattern is dependent on the randomness of the fibril structure (figure 7).We further separated the effects of randomness in fibril placement to randomness in fibril polarity by comparing four test cases, alternating polarity fibrils in a perfect lattice, alternating polarity fibrils with shifted lattice placement, random polarity fibril distribution in a perfect lattice, and random polarity fibrils with shifted lattice placements (figure 8). From this we observed that the random polarity of the fibrils had a far greater effect on the spatial emission profile than the exact fibril locations, which we attribute to the clustering of fibrils of single polarity. We found that increasing fibril density also results in increased *F*_SHG_/*B*_SHG_ ratios (figures 5(A)–(C)). Additionally, we showed that increasing the variation in fibril diameter results increased the *F*_SHG_/*B*_SHG_ values, suggesting that larger fibrils have a greater effect on the emission directionality even in the presence of smaller fibrils (figure 5(d)).

The wavelength dependence of the relative conversion efficiency has remained an unresolved issue. Several previous reports of the wavelength dependence of SHG from collagen have shown contradictory results either showing little spectral dependence [48, 58], a monotonic decrease with wavelength [59] or oscillatory responses [60]. While we found the conversion efficiency decreased with increasing wavelength over this range (∼3 fold) there was poor agreement with any two-state model based on possible resonances [35]. The nearest resonances for collagen are in the ∼200–220 nm range. However, the band around ∼360 nm that is associated with crosslinking has been invoked to explain the decreasing SHG response with wavelength [59]. However, the role of this transition is not obvious as while crosslinking can enhance the SHG conversion by increasing the local collagen density, the SHG is thought to arise from coherent amplification from peptide bonds [18]. In this computational model, we did not incorporate the two-state model, and we demonstrated a similar decrease in SHG conversion with increasing wavelength dependence in the SHG intensity (figure 7(B)) as previously determined experimentally. This computational analysis shows that the fibril architecture is sufficient to characterize the wavelength dependence of the SHG conversion efficiency in the 800–1200 nm spectral range. Moreover, this metric is less sensitive to intrinsic fibril size and packing than the emission directionality.

In addition to its use in simulation studies alone, our model has the potential to be combined with experiments from a dedicated new instrument that spatially images the emission pattern to determine fibril structure without the need for electron microscopy. A position sensitive system would then form the basis of SHG emission tomographic and complement our previously reported excitation tomographic approach to obtain true 3D fibrillar structure [61].

While providing detailed understanding of fibril architecture on the SHG spatial emission and conversion efficiency, there is also biological significance to the work. There is an associated collagen remodeling with all epithelial cancers, where these comprise ∼90% of all cancers. However, the form of this remodeling can have different manifestations, e.g. alterations of existing collagen or new collagen synthesis. Moreover, these can have different temporal patterns even in the same cancer type [62]. The detailed analysis here can provide new insight into disease etiology and progression, where this could be used both for ex vivo diagnostics but to also determine the effect of chemotherapies on the tissue structure.

## 7 Conclusions

We have developed a generalized 3D computational model based on QPM that can predict the spatial emission pattern and SHG conversion efficiency based on the underlying fibril size, packing and polarity. This modeling approach allows these predictions to be made on non-periodic fibril assemblies found in intact tissues. Using simulations to probe the parameter space allows us to dissect out the roles of these factors in determining the SHG creation attributes, and moreover allows us to place bounds on the fibril size, polarity and distribution in previously studied tendon and ovarian tissues. The extracted values are in good agreement known values from structural biology and also reproduce the SHG wavelength dependence without invoking a two-state model. This analysis provides further insight into the SHG contrast and also provides an additional means of differentiating normal and diseased tissues.

## Data Availability

All data that support the findings of this study are included within the article (and any supplementary files). Details of employed QPM method available at http://doi.org/10.1088/2515-7647/ae37b3/data1.
